# Generation of Charges During the Synthesis of Nanopowders of Doped Cerium Dioxide in Combustion Reactions

**DOI:** 10.3390/ma17246066

**Published:** 2024-12-12

**Authors:** Alexander Ostroushko, Olga Russkikh, Tatiyana Zhulanova, Anastasia Permyakova, Elena Filonova

**Affiliations:** 1 Institute of Natural Sciences and Mathematics, Ural Federal University, 620002 Yekaterinburg, Russia; alexander.ostroushko@urfu.ru (A.O.);; 2 Institute of High Temperature Electrochemistry of the Ural Branch of the Russian Academy of Sciences, 620137 Yekaterinburg, Russia

**Keywords:** ceria, electron microscopy, organic precursors, charge generation, nanomaterials

## Abstract

The development and characterization of synthesis techniques for oxide materials based on ceria is a subject of extensive study with the objective of their wide-ranging applications in pursuit of sustainable development. The present study demonstrates the feasibility of controlled synthesis of Ce_1−x_M_x_O_2−δ_ (M = Fe, Ni, Co, Mn, Cu, Ag, Sm, Cs, x = 0.0–0.3) in combustion reactions from precursors comprising glycine, polyvinyl alcohol, polyvinylpyrrolidone, polyethylene glycol, and cellulose as organic components. Controlled synthesis is achieved by varying the composition of the precursor, the type of organic component, and the amount of organic component, which allows for the influence of the generation of high-density electrical charges and outgassing during synthesis. The intensity of charge generation is quantified by measuring the value of the precursor–ground potential difference. It has been demonstrated that an increase in the intensity of charge generation results in a more developed morphology, which is essential for the practical implementation of ceria as a catalyst to enhance contact with gases and solid particles. The maximum value of the potential difference, equal to 68 V, is obtained during the synthesis of Ce_0.7_Ni_0.3_O_2−δ_ with polyvinyl alcohol in stoichiometric relations, which corresponds to a specific surface area of 21.7 m^2^ g^−1^. A correlation is established between the intensity of gas release for systems with different organic components, the intensity of charge generation, morphology, and the value of the specific surface area of the samples.

## 1. Introduction

The extensive and continuous research on oxide materials based on doped cerium dioxide has been driven by the potential applications of these materials, which span a wide range of fields. These include their primary use as components in catalysis [1,2,3,4,5,6] and for efficient purification of contaminated sources [1,7,8]. Additionally, they can be employed as constructional materials for energy conversion and storage devices [9,10,11], as well as in sensors [1,12], electrolytes for solid oxide fuel cells [13,14,15,16], the production of luminescent compositions [17], polishing additives [18,19], and biomedical applications [1,20,21,22,23,24]. To meet the specifications of the intended application, it is essential that the obtained ceria-based materials possess the requisite properties, including an optimal morphology [1,25,26,27,28].

According to the literature data, cerium dioxide can be obtained through a variety of methods, including the hydrothermal method [27,28,29,30], sol-gel methods [27,28,29], precipitating techniques [27,28,29], combustion synthesis [13,14,15,27,28,29], solid-state reaction [16,29,30], and the template method [31]. It should be noted that there are several challenges associated with these methods. For instance, the hydrothermal method necessitates the utilization of proprietary apparatus for the reaction vessels, which consequently impacts the yield based on the capacity of the equipment. To achieve increased yields, it may be necessary to either acquire additional equipment or utilize equipment with augmented capacities. Nevertheless, an increase in the volume of the reaction vessel may result in a concomitant reduction in the degree of control that can be exerted over the reaction process. The sol-gel method offers several advantages, including good control over the stoichiometry and homogeneity of the final product, as well as the ability to prepare nanostructured ceria materials [27,29]. However, the sol-gel process frequently necessitates meticulous regulation of the reaction parameters and may entail several stages, which can prove to be a significant time investment.

In principle, cerium dioxide can be successfully synthesized in reactions such as solution combustion synthesis. The high-temperature combustion process results in the formation of CeO_2_ nanoparticles with high crystallinity and purity [27]. The combustion method is an attractive option due to its simplicity, short reaction time, and capacity to produce large quantities of CeO_2_ nanomaterials. However, the capacity to regulate the morphology and size of the nanoparticles is limited, and the high-temperature combustion process may result in the agglomeration of the particles. Moreover, it is of great importance to regulate the characteristics of cerium dioxide particles, particularly regarding charge generation [32,33].

The precursor combustion processes during the synthesis of various oxide, chalcogenide and other functional materials are accompanied by electrochemical phenomena. Thus, the emergence of a potential difference on different sides of the combustion front during the implementation of processes of self-propagating high-temperature synthesis (SHS) has been discovered and described in research [34,35,36,37,38,39,40,41,42,43]. The main reasons for the appearance of potential differences ranging from a few hundredths to several volts include the spatial separation of charges of different signs due to their different mobility when passing the combustion front. The phenomenon of generation of electric charges during heating and combustion of organic–inorganic (mainly nitrate-containing) precursors [33,44,45,46,47,48,49,50] has been established in the processes of obtaining complex oxides such as solution combustion synthesis.

The generation of charges has been found to begin at the stage of heating the initial compositions even before the start of intense combustion (pyrolysis) [44,45]. The generation also continues during combustion, and the potential difference measured between the precursor and the ground reaches tens and even hundreds of volts [33,44,45,46,47,48,49,50]. As shown earlier on samples of precursors containing film polymers [44,45], the surface charge density can be more than 1 μC cm^−2^, and the volume density can be 2 × 10^15^ of charges per cm^3^. Charges in precursors appear due to the removal of charged molecular groups into the environment, which have the opposite sign of charge to the generated one [33,44,45,46,47,48,49,50]. It can be assumed that water molecules and carbonate-like groups that carry away an electron, i.e., are negatively charged, or nitrogen monoxide molecules, on the contrary, positively charged, can be released into the gaseous environment [44,45]. Moreover, for the generation of high-density charges in precursors, it is sufficient to have one charged molecular particle per several hundred thousand molecules of released low-molecular-weight compounds, which corresponds to a loss of 1% of the mass of the original composition [44,45]. Obviously, this is the reason why the generation of charges can already be recorded at the stage of heating the composition.

We have described the charge generation for reaction systems in which widely used materials based on lanthanum manganite with a perovskite-type structure [48], strontium hexaferrite (magnetoplumbite structure) and other types of complex oxides [33,47,49,51] were synthesized. The experimental ranking of the initial reaction systems with different organic substances and their relative amounts in terms of the intensity of charge generation allows for the selection of optimal options in terms of the properties of the resulting complex oxides [51,52]. The dependence of charge generation on the type and amount of dopant introduced into some complex oxides has also been established [33,50,52]. Mutual repulsion of synthesized nanoparticles of complex oxides due to the presence of similar charge leads to the emergence of an additional supply of surface energy, which during sintering of compacted complex oxides, provides a significant decrease in the temperature of intensive material shrinkage (sintering) [33,48,52]. This is also manifested in an increase in the specific surface area of powders obtained under conditions of intensive charge generation and with similar particle sizes compared to powders synthesized with low charges [52]. The possibility of controlling the sintering temperature and the achieved shrinkage of materials is an important aspect, for example, in the preparation of compositions consisting of different complex oxides in one sintering cycle, solid oxide fuel cells, including an oxygen conducting membrane and electrodes, catalytic compositions, etc. The stored excess surface energy of nanoparticles, due to their weaker contact during synthesis, dissipates during sintering in the form of released heat.

In [47,49,52], we have already demonstrated examples of optimized target properties of complex oxide compositions with the charge generation phenomenon, material texture and nanoparticle ensemble morphology, catalytic properties, electrical conductivity, specified phase composition, thermodynamic stability, etc. The influence on the properties of the resulting materials has been simultaneously demonstrated with the generation of charges by an external magnetic or electromagnetic field, which, in some cases, makes it possible to achieve an increase in magnetic properties due to the targeted creation of the texture of chain-like particle aggregates [51,52]. These examples confirm the potential for further research in the field of charge generation processes. There is reason to believe that the above processes can be used, among other things, to optimize the properties of functional complex oxide materials obtained as coatings directly on substrates [53,54,55,56,57,58,59,60,61,62,63,64,65,66,67].

It is also an urgent problem to control the properties of ceria powders taking into account charge generation [33]. In this work, we have carried out a more detailed study of the charge generation processes during the preparation of complex oxides based on doped ceria from nitrate-containing compositions, including organic polymer components of a different nature and a low-molecular-weight compound, glycine. An important point in the preparation and use of ceria-based materials is their phase composition, which has been studied for some systems by X-ray diffraction [68,69,70,71,72,73,74,75]. In some cases, particularly the use of complex oxides in catalysis, the production of fuel cell components, the presence of additional phases in the functional material, in addition to cerium dioxide, increases their target characteristics; therefore, options for homogeneous doping with the creation of solid solutions based on cerium dioxide, and heterogeneous doping with the emergence of heterogeneous systems are considered. The compositions synthesized in this work were chosen in such a way that some of them are solid solutions, as mentioned above, while others are at least two-phase. The influence of the presence of heterogeneity in complex oxide systems on charge generation is also interesting from a scientific and practical point of view.

When alkali metals (potassium, cesium) are introduced into precursors, at least up to their content in cerium dioxide of 0.3 molal units, solid solutions with a fluorite-like structure are formed [33]. The introduction of iron [33] and samarium [76] also leads to the formation of solid solutions, in contrast to systems containing nickel, copper and silver, where, in addition to the cerium dioxide phase, impurities of NiO [33], CuO [33] and metallic silver are formed [33,77]. Manganese doping of ceria is observed up to 0.2 molal units [33]. In this work, three types of systems have been studied, including dopants of a different nature, with the formation of single-phase solid solutions (Fe, Sm, Cs), with oxide and metal impurity particles (Ni, Cu, Ag). Among the systems with oxide impurities, the nickel-doped ceria was chosen for a more detailed study since this complex oxide is more stable compared to copper- and silver-doped compounds and is not subject to reduction during synthesis.

## 2. Materials and Methods

### 2.1. Preparation of the Materials

For the synthesis of the studied complex oxide compositions, the following reagents of not less than “reagent grade” were used as starting salts: hexahydrate cerium nitrate (III) Ce(NO_3_)_3_·6H_2_O; nickel nitrate, manganese, copper and iron in the form of the corresponding crystallohydrates, Ni(NO_3_)_3_·6H_2_O, Mn(NO_3_)_3_·6H_2_O, Cu(NO_3_)_3_·3H_2_O and Fe(NO_3_)_3_·9H_2_O; and cesium and silver nitrates CsNO_3_, AgNO_3_. The nitrates were dissolved in distilled water, mixed with each other in specified stoichiometric proportions, and then with a solution of one of the selected organic components, polyvinyl alcohol PVA (of medium molecular weight with a viscosity of a 4% aqueous solution at 25 °C of 11 Pa s and the number of unsaponified acetate groups not more than 2%), polyvinylpyrrolidone PVP (molecular weight 40000, Sigma-Aldrich (St. Louis, MO, USA), PVP40), polyacrylamide PAA (molecular weight 3·10^5^ Sigma-Aldrich (St. Louis, MO, USA)), polyethylene glycol PEG (molecular weight 5000 Fluka Chemika (Buchs, Switzerland)), glycine Gly ‘chemically pure’ qualification. Samarium was taken in the form of oxide Sm_2_O_3_ ‘chemically pure’, which was previously dissolved in chemically pure nitric acid HNO_3_. Cellulose (C) was used in the form of ashless paper filters Filtrak (Baerenstein, Germany), while impregnating them with moisture-holding capacity with salt solutions.

For convenience, several complex oxide materials and bulk compositions were designated: CeO_2_, Ce_0.9_Fe_0.1_O_2−δ_ (Fe01), Ce_0.7_Fe_0.3_O_2−δ_ (Fe03), Ce_0.9_Ni_0.1_O_2−δ_ (Ni01), Ce_0.7_Ni_0.3_O_2−δ_ (Ni03), Ce_0.9_Mn_0.1_O_2−δ_ (Mn01), Ce_0.7_Mn_0.3_O_2−δ_ (Mn03), Ce_0.9_Ag_0.1_O_2−δ_ (Ag01), Ce_0.9_Cs_0.1_O_2−δ_ (Cs01), Ce_0.8_Sm_0.2_O_2−δ_ (Sm02). The stoichiometric ratio of the organic component, as the reducing agent in the combustion reactions, and the nitrate part of the salts, as the oxidizing agent, was calculated with the equations of the corresponding reactions, formally taking carbon dioxide, water, and molecular nitrogen as the released gaseous products. The actual ratio used for the synthesis of complex oxides was as follows:

(1) $$\varphi =\frac{m_{1}}{m_{stoich}},$$

where *m*_1_ is the weight of the organic component taken to prepare the composition; *m_stoich_* is the stoichiometric weight of the organic component calculated from the above reaction equations. Values of *φ* > 1 mean that the organic component has been taken in excess. It should be noted that the actual combustion process of the compositions differs somewhat from the formal reaction scheme because; in addition to the above-mentioned gaseous substances, a quantity of carbon monoxide and nitrogen oxides is released during the synthesis of oxide materials [31,46,48,49,50].

Complex oxides in combustion reactions of the initial compositions were synthesized in a porcelain cup heated by a quartz electric heater with a thermostat with a heating time to operating temperature of 30 s after the water was removed directly during heating or by drying at room temperature (298 К). The spiral heating element of the heater generated an alternating magnetic field with a frequency of 50 Hz [31,48], with a magnetic component strength of 8 μT and an electric component of about 1 kV m^−1^. Such a field, as was shown previously [31], shifted the potential difference between the ground and the precursor proportionally towards positive values compared to the purely thermal effect.

### 2.2. Methods

During heating and combustion, the potential difference between the precursor and the ground was measured with an IPEP-1 electrostatic field parameter meter by using a computer recording of the results. The readings were taken from a special metal screen connected to a heat-resistant electrode placed in the reaction medium. The magnitude of the measured potential difference correlated with the magnitude of the charges accumulated by the precursors. The difference between the measured value for a specific precursor and the value of the potential difference for a blank experiment with heating in the measuring system of the electrode itself was taken as the value of the potential difference discussed below.

The combustion temperature of the compositions was recorded with a Testo 835-T2 infrared pyrometer (Testo AG, Titisee-Neustadt, Germany). The composition of the gases during the combustion of the compositions with the determination of the concentration of carbon monoxide CO, and nitrogen oxides NO and NO_2_ was monitored with a Testo 350 XL gas analyzer (Testo AG, Titisee-Neustadt, Germany). The final heat treatment of the samples was carried out in air at 650 °C for 48 h. The morphological features of the obtained complex oxide samples were illustrated by the method of scanning electron microscopy: AURIGA CrossBeam microscope with local energy-dispersive microanalysis and EDX system (Carl Zeiss NTS, Oberkochen, Germany). The work was controlled, and the obtained data were analyzed with the software package Analysis Station, AURIGA series, version 3.7.

The phase composition of the obtained powders after their final heat treatment at 650 °C for 48 h was determined using a Bruker D8 Advance diffractometer (Bruker, Karlsruhe, Germany). X-ray patterns were recorded in CuKα radiation (λ = 1.5418 Å) in the range of angles 20° ≤ 2Θ ≤ 80° at a speed of 2° per minute. The crystal structure was calculated using the Fullprof software, version 8.10. The specific surface area was measured by the method of low-temperature nitrogen sorption with thermal desorption using the TriStar 3020 automated sorption unit (Micromeritics, Norcross, GA, USA).

## 3. Results and Discussion

### 3.1. Phase Composition and Electron Microscopy

**Figure 1:**
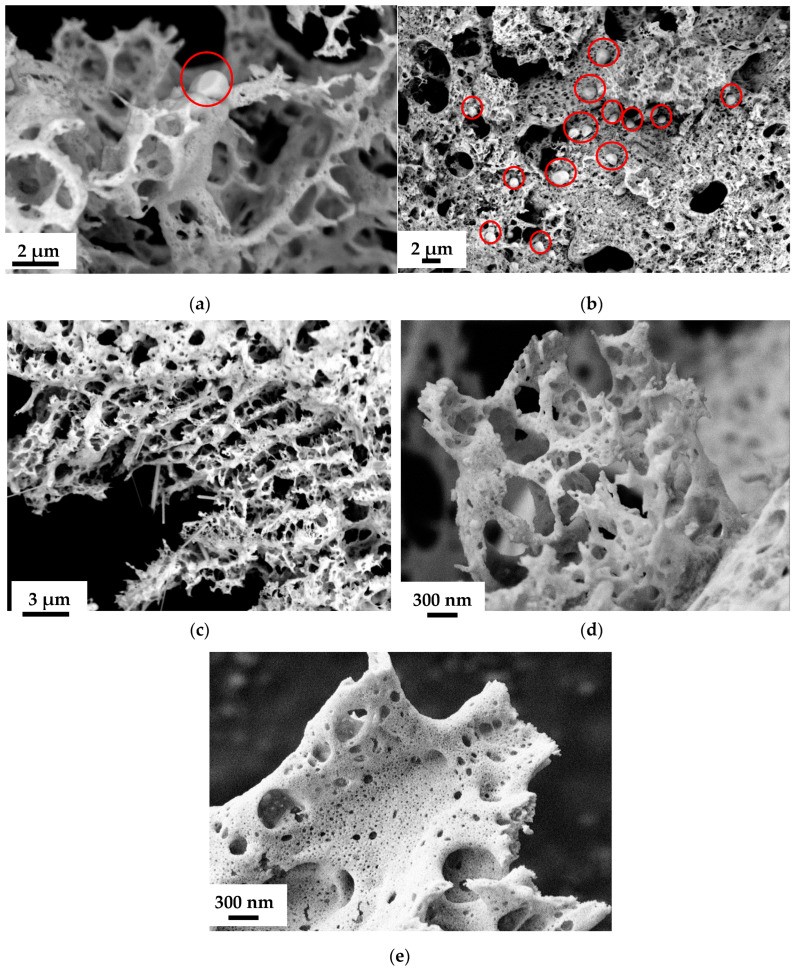
SEM images of oxide materials synthesized from precursors containing polyvinyl alcohol at different amounts relative to the stoichiometric (*φ*) for the combustion reaction, bulk composition: Ce_0.9_Ag_0.1_O_2−δ_ (*φ* = 1) (**а**); Ce_0.9_Ag_0.1_O_2−δ_ (*φ* = 2) (**b**); Ce_0.9_Cu_0.1_O_2−δ_ (*φ* = 1) (**c**); Ce_0.9_Ni_0.1_O_2−δ_ (*φ* = 1) (**d**); Ce_0.9_Fe_0.1_O_2−δ_ (*φ* = 1) (**e**).

**Figure 2:**
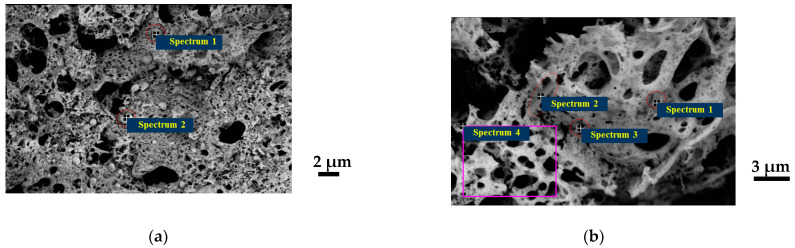
SEM images with EDX analysis for Ce_0.9_Ag_0.1_O_2−δ_ (**а**) and Ce_0.9_Ni_0.1_O_2−δ_ (**b**) synthesized from precursors containing polyvinyl alcohol (*φ* = 1) (purple indicates the region where the second phase of NiO is absent).

**Figure 3:**
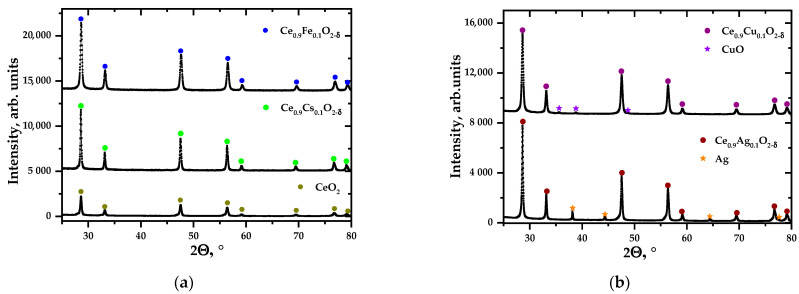
Examples of XRD of single-phase samples (**a**) and two-phase samples (**b**).

As mentioned above, complex oxide systems formed in combustion reactions with charge generation have a highly developed surface due to the mutual repulsion of nanoparticles. The resulting ensembles of particles with hierarchical organization are suitable for direct use in catalysis because they provide good contact with gases and solid particles [31], including soot. To make what we are discussing clear, we illustrated their morphology with electron micrographs with EDX analysis (Figure 1 and Figure 2, Table 1) and their phase composition (Figure 3, Table 2). Another important point when discussing systems where the synthesis results in non-single-phase products is the state of the impurity phases, which is also well illustrated by scanning electron microscopy (SEM). The micrographs (Figure 1) and the results of EDX analysis (Figure 2) show that metallic silver, which is not present in cerium dioxide, forms round or hemispherical particles that appear lighter in SEM photographs (red circles, Figure 1 and Figure 2), as do nickel oxide particles. Some of the silver is distributed over the surface of the ceria in the form of even more highly dispersed nano-sized particles, which can be detected by elemental analysis (Figure 2). The copper oxide particles are in the form of whiskers (Figure 2). For comparison, Figure 1 shows a snapshot of a single-phase sample obtained in a system containing iron-doped ceria, where round particles are visible. For a more detailed study of the charge generation processes, at this stage, a system containing nickel was selected from the systems containing oxide impurity particles. This choice is related to the similarity in the morphology of the main and impurity phase particles in all the samples discussed.

### 3.2. Synthesis Characteristics

**Figure 4:**
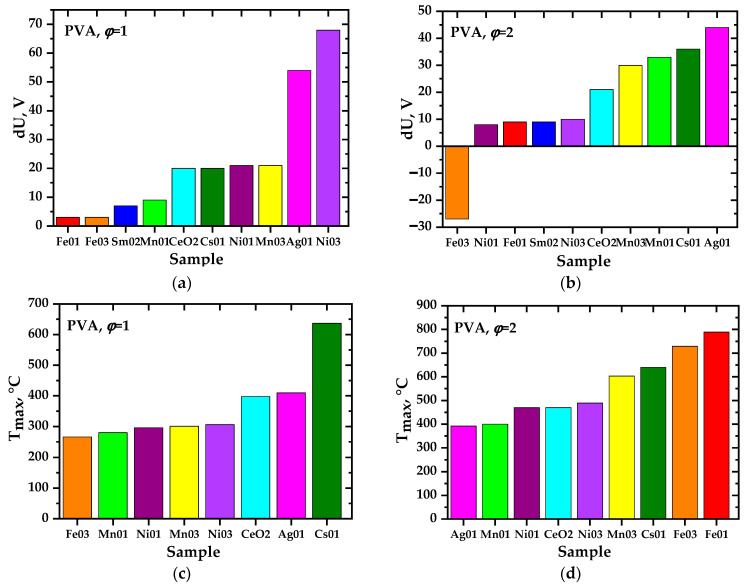
Maximum potential difference (**a**,**b**) and maximum temperature (**c**,**d**) obtained during the synthesis of the complex oxide material Ce_1−x_M_x_O_2−δ_ (bulk composition) from precursors containing polyvinyl alcohol with *φ* = 1 (**a**,**c**) and with *φ* = 2 (**b**,**d**). The diagrams show the doping elements, M = Fe, Mn, Ni, Ag, Cs, and their mole fractions (0.1; 0.3).

**Figure 5:**
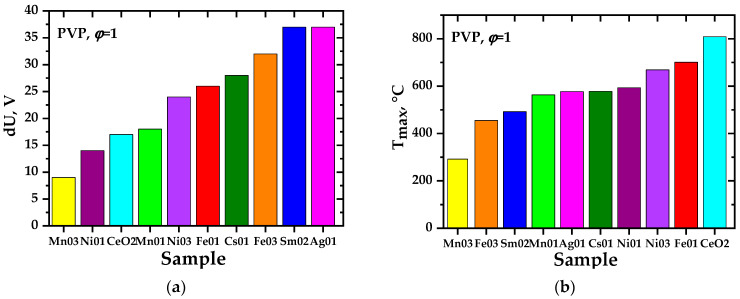
Maximum precursor–ground potential difference (**a**) and maximum temperature (**b**) obtained during the synthesis of the complex oxide material Ce_1−x_M_x_O_2−δ_ (bulk composition) from precursors containing polyvinylpyrrolidone with *φ* = 1.

**Figure 6:**
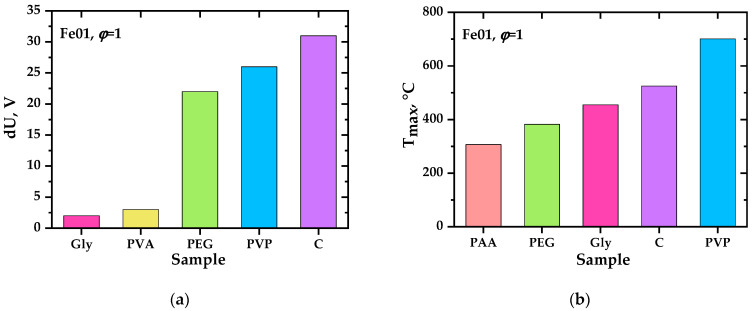
Maximum precursor–ground potential difference (**a**) and maximum temperature (**b**) obtained during the synthesis of the complex oxide material Ce_0.9_Fe_0.1_O_2−δ_ from precursors containing glycine (Gly), polyvinyl alcohol (PVA), polyethylene glycol (PEG), polyvinylpyrrolidone (PVP), cellulose (C), polyacrylamide (PAA) with *φ* = 1.

**Figure 7:**
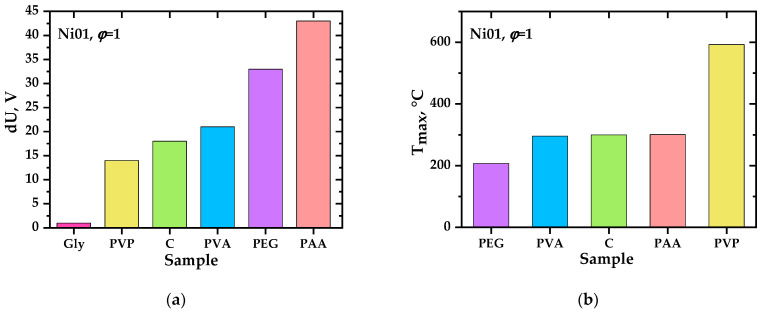
Maximum precursor–ground potential difference (**a**) and maximum temperature (**b**) obtained during the synthesis of the complex oxide material Ce_0.9_Ni_0.1_O_2−δ_ from precursors containing glycine (Gly), polyvinyl alcohol (PVA), polyethylene glycol (PEG), polyvinylpyrrolidone (PVP), cellulose (C), polyacrylamide (PAA) with *φ* = 1.

As a result of experimental studies, it was found that in almost all precursors intended to produce oxide systems based on ceria, the charges initially discovered [31] are generated, as in perovskite systems [46]. The presence of the second phase in ceria does not prevent their formation; on the contrary, in some cases, it favors it. This conclusion is important, at least for the practical application of the charge generation phenomenon. The studied systems are ranked according to the values of the achieved precursor and ground potential differences (intensity of charge generation). Figure 4, Figure 5, Figure 6 and Figure 7 show the values of the maximum achieved charges and temperatures in the precursors depending on their composition in terms of the elements introduced, the nature of the organic reagent, and the ratio of organic and nitrate parts (oxidizing agent). In this case, certain patterns can be seen that are important both for understanding the processes of charge generation and from the point of view of practical selection of conditions for the synthesis of complex oxides to achieve their required properties, in particular, sintering parameters.

In this system, to produce cerium dioxide containing 0.3 mol units of iron, in contrast to other systems, a reliable generation of negative charges was recorded despite the influence of the electromagnetic field of a spiral heater [46,48], which stimulates the emission of electrons by precursor particles. A relative decrease in positive charge values for systems based on ceria compared to those based on perovskite [46], including their shift to the negative region, may be due to the fact that in the resulting oxides, the main transition metal (its sublattice) tends to increase the effective oxidation state (from Mn^3+^ to Mn^4+^) for manganese when doping the lanthanum sublattice, and, in contrast, to decrease it for cerium, which is in the maximum oxidation state (4+). These are multidirectional processes of oxidation or reduction of transition metal ions, which, among other things, can influence the tendency to the formation and emission of charged particles; manganese is negative, as is cerium. At the same time, in the real manganite structure, under the conditions studied, there is usually an excess of oxygen, and in cerium dioxide, there are oxygen vacancies. It is also characteristic that undoped lanthanum manganite shows maximum charges in the positive region, which cannot be said for pure cerium dioxide in comparison to doped systems.

The largest precursor–ground potential difference for systems containing the polymeric components PVA and PVP (*φ* = 1,2) occurred during the synthesis of a non-single-phase material with a bulk composition of Ce_0.9_Ag_0.1_O_2−δ_, where the presence of metallic silver was detected in addition to the cerium dioxide phase. Apparently, the presence of highly conductive metallic nanoparticles promotes the generation of charges in the precursors, thereby facilitating the release of electrons. Considering the small free path of electrons in the reaction gas medium, not exceeding a few micrometers, and their interaction with molecules, negatively charged molecular particles (groups), which we discussed above, are eventually released. It can be assumed that if the generation of charges is intensified by the presence of metal or other conducting nanoparticles, the preservation of the resulting charges in the precursor should, on the contrary, be facilitated by the low conductivity of the matrix or, in the limiting case, by the dielectric properties.

In the preparation of single-phase ceria-based oxide materials, namely, Ce_0.9_Cs_0.1_O_2−δ_, Ce_0.9_Fe_0.1_O_2−δ_, Ce_0.7_Fe_0.3_O_2−δ_, Ce_0.9_Mn_0.1_O_2−δ_, и Ce_0.8_Sm_0.2_O_2−δ_, a higher potential difference was observed during the synthesis from precursors containing PVP (*φ* = 1), while the resulting precursor–ground potential difference for precursors containing PVA (*φ* = 1) was generally higher when obtaining heterogeneous samples, Ce_0.9_Ag_0.1_O_2−δ_, Ce_0.7_Mn_0.3_O_2−δ_, Ce_0.9_Ni_0.1_O_2−δ_, Ce_0.7_Ni_0.3_O_2−δ_. There is a correlation between the morphology of the resulting powders (Figure 1) and the charges recorded in them. The samples in which higher charges were generated during synthesis had a more developed spatial structure of particle ensembles. For example, iron-doped ceria powders obtained from compositions containing PVA could be characterized by relatively low charges (Figure 4), and in SEM images (Figure 1), they appear denser compared to other samples.

**Figure 8:**
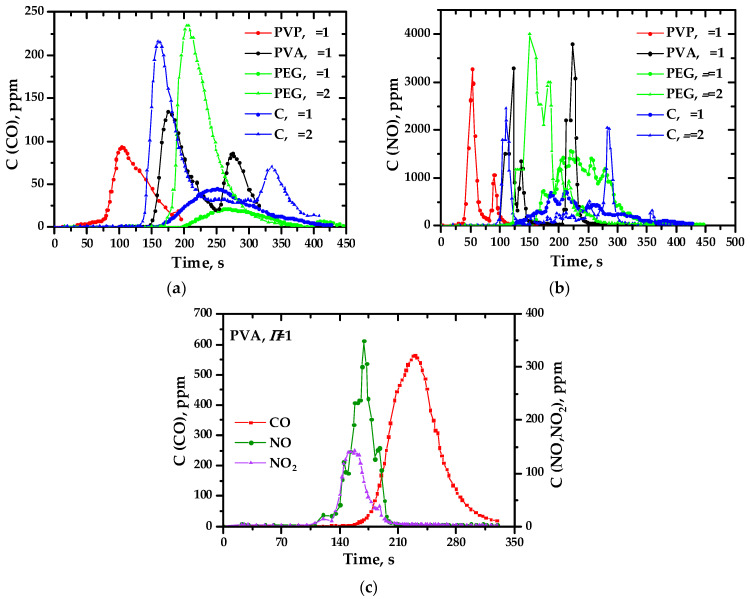
Concentration profiles of the release of CO (**a**) and NO (**b**) during the synthesis of Ce_0.9_Ni_0.1_O_2−δ_ (bulk composition) from precursors containing polyvinylpyrrolidone (PVP) with *φ* = 1, polyvinyl alcohol (PVA) with *φ* = 1, polyethylene glycol (PEG) with *φ* = 1, 2, cellulose (C) with *φ* = 1, 2; CO, NO, NO_2_ (**c**) during the synthesis of Ce_0.7_Ni_0.3_O_2_ (bulk composition) from a precursor containing polyvinylpyrrolidone with *φ* = 1.

**Figure 9:**
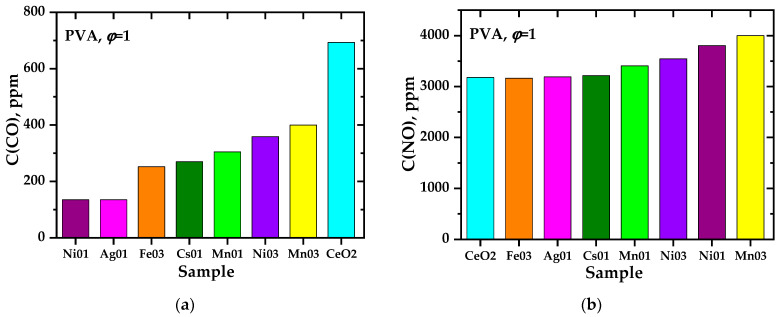
Maximum concentration of CO (**a**) NO (**b**) during the synthesis of materials with bulk composition Ce_1−x_M_x_O_2−δ_ obtained from precursors containing polyvinyl alcohol with *φ* = 1. The diagrams show the doping elements, M = Fe, Mn, Ni, Ag, Cs, and their mole fractions (0.1; 0.3).

Analysis of the data obtained from the point of view of the influence of various factors on the charge generation processes showed that, regarding the synthesis of perovskite complex oxide systems [46], there is no direct correlation between the maximum combustion temperature of precursors and the intensity of charge generation. Figure 8 and Figure 9 show the most characteristic profiles of the release of gaseous products during the combustion of precursors and the maximum concentrations of gaseous components achieved. As regards the system based on LaMnO_3±y_ [46], a time shift between the maxima of the release of nitrogen oxides and carbon monoxide (non-synchronous release of gases of different compositions) was recorded, apparently related to the staged nature of the pyrolysis (combustion) process. Phenomenological observations have confirmed this aspect.

During the synthesis of such oxide compounds as CeO_2_, Ce_0.9_Cs_0.1_O_2−δ_, Ce_0.8_K_0.1_Cs_0.1_O_2−δ_, which have a white or light-yellow color, the stages of combustion (stepwise process) and the movement of the combustion front along the surface of the precursor were visually observed. The first stage, accompanied by the release of more nitrogen oxides, ended with the formation in the cup of a dark mass with a flat surface containing an incompletely burnt carbonaceous part, which burned out in the second stage during further heating with the formation of a light powder of cerium oxide. The second stage of combustion was accompanied by the release of significant amounts of carbon monoxide along with carbon dioxide (as shown in previous studies [31,43]).

**Figure 10:**
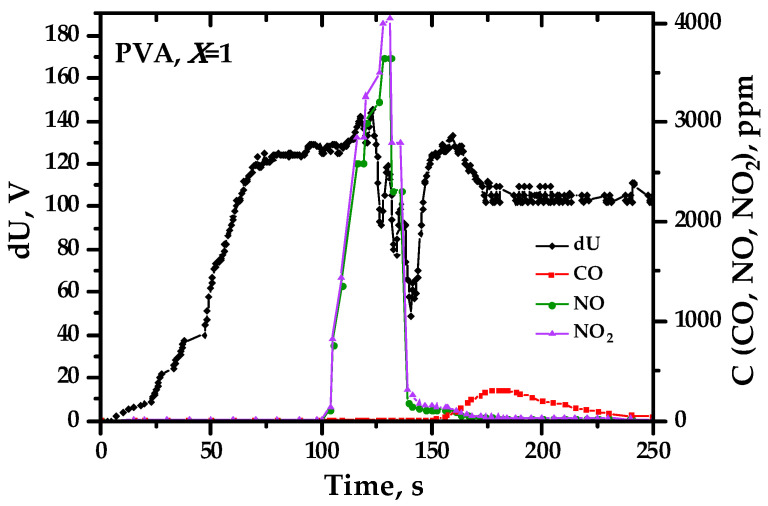
The precursor–ground potential difference and the concentration of gases released during the synthesis of the complex oxide Ce_0.9_Cs_0.1_O_2−δ_ from a precursor containing polyvinyl alcohol with *φ* = 1 (the data here are given without considering the blank experiment).

Figure 10 clearly illustrates the relationship between the generation of charges and the intensity of the ongoing process of releasing substances from the precursor into the environment. Here, a combined time profile of the precursor–ground potential difference and gas concentrations during the synthesis of Ce_0.9_Cs_0.1_O_2−δ_ oxide is shown. During heating to temperatures below the onset of combustion (on average 100–130 °C for different systems, depending on the composition of the resulting complex oxide), the precursor–ground potential difference increases. This is apparently related to the removal of negative charges by ionized water molecules, which are predominantly released during this period, according to thermal analysis data combined with mass spectrometry [31,43]. Then, when the temperature of the onset of combustion is reached, there is an intense release of nitrogen oxides, some of which can leave with a positive charge. This corresponds to a decrease in the existing potential and charge difference (apparently down to negative values, see Figure 4). Further intense release of carbon oxides with the creation of a quantity of entrained carbonate-like negatively charged particles again leads to an increase in the value of the potential difference. This confirms the previously proposed idea of charge generation by the entrainment of suitably charged particles.

During the experiments, it was found that for most of the ceria-based compositions studied, the concentration of nitrogen oxides is generally lower, and carbon monoxide is higher when the oxides are synthesized from organonitrate compositions containing an excess of the organic component. It should also be noted that the combustion temperature was higher for compositions containing an excess of the polymer component.

It can be assumed that the resulting complex oxide material acted as a combustion catalyst during the synthesis process, which is typical for ceria-based materials [31,46,78,79,80,81] and can be confirmed by experiments, the results of which are presented below. On the one hand, at a higher synthesis temperature, the residual carbon burned more intensely in the second stage of pyrolysis; so, a peak increase in the concentration of carbon oxides in the gases was recorded. On the other hand, the complex oxide material also catalytically influenced the reduction reaction of nitrogen oxides with carbon, carbon monoxide to molecular nitrogen, which is also recorded for doped cerium dioxide. A similar effect of the complex oxide material itself on the concentration of released nitrogen oxides and carbon monoxide was discovered for a system based on lanthanum manganite [46], which also has catalytic properties.

Thus, by adjusting the composition of the initial precursor, it is possible to simultaneously influence both the charge generation processes and the composition of the gaseous environment generated during the combustion process. This, in turn, influences the morphology and sintering parameters of the resulting complex oxides (mainly through charges), as well as the redox properties of the medium, which can determine the creation of the most favorable material for achieving the targeted material properties [45,47] of the defect structure of the complex oxide. In the abovementioned works, the double perovskite Sr_2_(Ni,Mg)MoO_6−δ_ was synthesized, which is promising as an anode of a solid oxide fuel cell [82,83]. The optimal properties of the samples were obtained during the synthesis from organic nitrate precursors containing a 3-fold excess of glycine, in which high charges and a reducing atmosphere were generated, which ensured the creation of the morphology and a given phase composition of the material with oxygen vacancies (thermodynamically stable in a reducing environment) and optimal electrical conductivity considering its defect structure.

### 3.3. Specific Surface Area

The relationship between the generation of charges and such a characteristic of the resulting complex oxide materials as the specific surface area, which, in turn, is related to the temperature of intensive sintering [31,46,50], was previously established with samples of two different materials, cerium dioxide, in which case iron was the dopant, and lanthanum manganite [50]. The same tendency of these materials to increase the specific surface area is shown when they are synthesized from compositions that ensure the occurrence of a larger potential difference (Table 3).

In the observed case, the complex oxide nanoparticles have similar sizes (Figure 1). Consequently, one of the main factors determining the specific surface area may be the degree of contact of these particles achieved during synthesis. It can be assumed that the generation of charges on the resulting nanoparticles, which leads to their mutual repulsion, provides an increase in the effective specific surface area, which, in turn, allows for further increase in the charges, and these factors act synergistically.

## 4. Conclusions

By studying the processes of synthesis of complex oxides based on cerium dioxide including Ce_1−x_M_x_O_2−δ_ (M = Fe, Ni, Co, Mn, Cu, Ag, Sm, Cs, x = 0.0−0.3) in combustion reactions of organic and inorganic compositions, the following has been found. Ranking of the initial compositions in terms of the intensity of charge generation depending on the nature of dopants, organic components and their relative amounts, and the phase composition of the product showed that the presence of phase impurities in a complex oxide does not prevent the generation of charges. The conditions for the generation of charges were favorable with the appearance of highly conductive metallic silver particles, which accelerated the emission of electrons. In the preparation of single-phase complex oxide materials (solid solutions), a higher potential difference was more often recorded during the synthesis from precursors containing polyvinylpyrrolidone. More intense charge generation accompanied the synthesis of heterogeneous products from compositions containing polyvinyl alcohol.

The generation of charges correlated with an intense release of gaseous combustion products (CO, NO, NO_2_), increasing the likelihood of molecular charged particles of one sign or another being carried into the environment. It is shown that both positive and negative charges can be generated in precursors during combustion. There is no obvious correlation between the combustion temperature of precursors and the generation of charges in them. Intense charge generation leads to the production of less dense complex oxide powders synthesized under such conditions with a developed spatial structure. Such materials are interesting from the point of view of their use in catalysis, as components of solid oxide fuel cells, especially since their synthesis in the form of coatings applied to the support is quite feasible in practical applications in combustion reactions.

## Figures and Tables

**Table 1:** Results of EDX analysis of the samples Ce_0.9_M_0.1_O_2−δ_ synthesized from precursors containing polyvinyl alcohol (*φ* = 1).

Sample	Spectrum on Figure 2	Elements, at. %			
		C	O	Ag	Ce
Ce_0.9_Ag_0.1_O_2−δ_	1	6.73	23.60	66.74	2.93
	2	10.36	28.06	53.10	8.47
Ce_0.9_Ni_0.1_O_2−δ_	1	17.15	46.41	35.28	1.16
	2	22.40	46.05	27.54	4.00
	3	25.56	50.38	8.04	16.03
	4	20.56	52.11	2.07	25.27

**Table 2:** Phase composition of samples synthesized with PVA.

Sample (Notation)	*φ* = 1	*φ* = 2
CeO_2_	CeO_2_	CeO_2_
Ce_0.9_Fe_0.1_O_2−δ_ (Fe01)	Ce_0.9_Fe_0.1_O_2−δ_	Ce_0.9_Fe_0.1_O_2−δ_
Ce_0.7_Fe_0.3_O_2−δ_ (Fe03)	Ce_0.7_Fe_0.3_O_2−δ_	Ce_0.7_Fe_0.3_O_2−δ_
Ce_0.9_Ni_0.1_O_2−δ_ (Ni01)	Ce_0.9_Ni_0.1_O_2−δ_, NiO	Ce_0.9_Ni_0.1_O_2−δ_, NiO
Ce_0.7_Ni_0.3_O_2−δ_ (Ni03)	Ce_0.7_Ni_0.3_O_2−δ_, NiO	Ce_0.7_Ni_0.3_O_2−δ_, NiO
Ce_0.9_Mn_0.1_O_2−δ_ (Mn01)	Ce_0.9_Mn_0.1_O_2−δ_	Ce_0.9_Mn_0.1_O_2−δ_
Ce_0.7_Mn_0.3_O_2−δ_ (Mn03)	Ce_0.7_Mn_0.3_O_2−δ_, Mn_2_O_3_, MnO_2_	Ce_0.7_Mn_0.3_O_2−δ_, Mn_2_O_3_, MnO_2_
Ce_0.9_Ag_0.1_O_2−δ_ (Ag01)	Ce_0.9_Ag_0.1_O_2−δ_, Ag	Ce_0.9_Ag_0.1_O_2−δ_, Ag
Ce_0.9_Cs_0.1_O_2−δ_ (Cs01)	Ce_0.9_Cs_0.1_O_2−δ_	Ce_0.9_Cs_0.1_O_2−δ_
Ce_0.8_Sm_0.2_O_2−δ_ (Sm02)	Ce_0.8_Sm_0.2_O_1.9_	Ce_0.8_Sm_0.2_O_2−δ_
Ce_0.9_Cu_0.1_O_2−δ_ (Cu01)	Ce_0.9_Cu_0.1_O_2−δ_, CuO	Ce_0.9_Cu_0.1_O_2−δ_, CuO

**Table 3:** Specific surface area of studied samples.

Sample	S_sp_, m^2^ g^−1^
Ce_0.9_Fe_0.1_O_2−δ_ (PVA, *φ* = 2)	32.4
Ce_0.9_Fe_0.1_O_2−δ_ (PVP, *φ* = 2)	11.8
Ce_0.9_Fe_0.1_O_2−δ_ (C, *φ* = 1)	20.9
Ce_0.9_Fe_0.1_O_2−δ_ (C, *φ* = 2)	11.3
Ce_0.9_Fe_0.1_O_2−δ_ (Gly, *φ* = 1)	8.9
Ce_0.9_Fe_0.1_O_2−δ_ (Gly, *φ* = 2)	18.5
Ce_0.9_Fe_0.1_O_2−δ_ (PEG, *φ* = 1)	21.6
Ce_0.9_Fe_0.1_O_2−δ_ (PEG, *φ* = 2)	9.6
Ce_0.9_Ag_0.1_O_2−δ_ (PVA, *φ* = 1)	7.8
Ce_0.9_Ag_0.1_O_2−δ_ (PVA, *φ* = 2)	9.7
Ce_0.9_Ag_0.1_O_2−δ_ (PVP, *φ* = 2)	8.0
Ce_0.9_Cu_0.1_O_2−δ_ (PVA, *φ* = 1)	16.5
Ce_0.9_Cu_0.1_O_2−δ_ (PVA, *φ* = 2)	14.0
Ce_0.9_Cu_0.1_O_2−δ_ (PVP, *φ* = 2)	26.0
Ce_0.7_Mn_0.3_O_2−δ_ (PVA, *φ* = 1)	23.0
Ce_0.7_Mn_0.3_O_2−δ_ (PVA, *φ* = 2)	18.7
Ce_0.9_Ni_0.1_O_2−δ_ (PVA, *φ* = 2)	20.9
Ce_0.9_Ni_0.1_O_2−δ_ (PEG, *φ* = 2)	14.3
Ce_0.7_Ni_0.3_O_2−δ_ (PVA, *φ* = 1)	21.7

## Data Availability

The original contributions presented in this study are included in the article. Further inquiries can be directed to the corresponding author.
